# A practical introduction to wavelet analysis in electroretinography

**DOI:** 10.1007/s10633-025-10070-x

**Published:** 2025-12-15

**Authors:** Yousif J. Shwetar, David S. Lalush, Alice Y. Zhang, J. Jason McAnany, Brett G. Jeffrey, Melissa A. Haendel

**Affiliations:** 1 Joint Department of Biomedical Engineering, University of North Carolina and North Carolina State University, Chapel Hill, NC USA; 2 Department of Ophthalmology, University of North Carolina at Chapel Hill, Chapel Hill, NC USA; 3 Department of Ophthalmology and Visual Sciences, University of Illinois Chicago, Chicago, IL USA; 4 Ophthalmic Genetics and Visual Function Branch, National Eye Institute, NIH, Bethesda, MD USA; 5 Department of Genetics, University of North Carolina, Chapel Hill, NC USA

**Keywords:** Electroretinography, Signal processing, Fourier transform, Wavelet transform, Diagnostics, Metrics

## Abstract

**Purpose:**

To provide a conceptual understanding of the continuous and discrete wavelet transforms (CWT, DWT) for clinical electroretinography (ERG) analysis, and how these methods uncover time–frequency features that complement traditional time-domain analysis.

**Methods:**

A technical overview without the use of mathematical formula describing the basics of CWT and DWT and implementation considerations. We also review an example of four standard ISCEV full-field ERG (ffERG) recordings from a healthy 32-year-old male.

**Results:**

Wavelet analysis uncovered time–frequency signatures absent in raw traces. In DA 0.01 cd s/m^2^ DWT scalogram, energy localized in the 2–5 Hz range, with CWT scalograms corroborating these findings. In DA 3.0 cd s/m^2^, a broader frequency response is seen across 10, 20 and 40 Hz center frequencies. A similar progression was found in the LA 3.0 cd s/m^2^, with additional low energy indices at 80 and 160 Hz. For the LA 30 Hz flicker, all frequency and time–frequency profiles effectively replicated the 30 Hz response of the cone system.

**Conclusions:**

CWT and DWT provide complementary and objective insight into ERG responses. Open-source MATLAB toolkit and step-by-step tutorial provided herein lower technical barriers and enable use by the broader community.

## Introduction

The electroretinogram (ERG) measures the change in voltage across the retina in response to a light stimulus, with clinical ERG analysis typically performed in the time domain (e.g., amplitude, implicit time, waveform shape) [1, 2]. However, the ERG is a complex signal with contributions from multiple cellular sources, and conducting analysis purely in the time-domain may limit additional valuable insight that time–frequency measures can offer. Many fields of electrophysiology including electrocardiology (ECG) and electroencephalography (EEG) have successfully leveraged wavelet transforms (WTs) to enhance interpretability and classification of biosignals. In ECG, discrete WT (DWT) has improved arrhythmic beat detection accuracy beyond conventional time-domain analysis (99.2% by He et al. [3], and 97.8% by Martis et al. [4]), while in EEG, it has enabled precise recognition of emotional and sleep states by capturing transient frequency-band activity (95% by Bajada and Borg [5], 93% by Singh et al. [6]).

Similar successes have been achieved in the assessment of ERG recordings;  Gauvin and colleagues applied wavelet analysis to isolate the ON and OFF components of the photopic ERG across a range of recording protocols (variations in stimuli strength and duration), linking wavelet descriptors to bipolar-cell function. Specifically, they found the ON and OFF bipolar cell responses to largely occupy the 20 and 40 Hz ranges of the photopic b-wave respectively, forming a ratio termed 40b:20b that effectively assessed the balance of these two cellular populations [7]. In another study of theirs, they used a similar approach to measure the relative energy of the oscillatory potentials (OPs) in the photopic ERG, and found that certain pathological conditions exhibit characteristic energy contributions in these high-frequency oscillations [8].

These applications have even been extended in the tasks of classifying neuropsychiatric conditions such autism spectrum disorder (ASD) [9] and attention deficit hyperactivity disorder (ADHD) [10]. Manjur et al. used DWT-based spectral energy features from ERGs to train ensemble machine learning (ML) models, achieving a balanced accuracy of ~ 81% when differentiating ASD, ADHD, and control groups, outperforming time-domain feature models by more than 10% [11]. WTs are objective, not based on a single time point in the waveform, can be resistant to the effects of noise, and have quick computational implementation. However, the technique has not been widely adopted in the field to date, in part due to perceived challenges in implementation.

Here, we provide a brief technical overview without the use of mathematical formulations of the WT as applied to standard ISCEV ERGs [1]; readers interested in mathematical formulation can refer to the Appendix. We describe the continuous wavelet transform (CWT) and DWT, and provide example application in ERGs recorded from a healthy human subject. To support implementation, we also provide openly accessible MATLAB (Natick, MA) code (available on GitHub) that performs the CWT and DWT operations demonstrated here. By providing a conceptual explanation and highlighting the capacity of the CWT and DWT to reveal specific time–frequency detail, we offer a practical, readily implementable framework for leveraging wavelet analysis in ERG research and clinical interpretation.

## A high-powered mathematical microscope: continuous wavelet transform

**Fig. 1:**
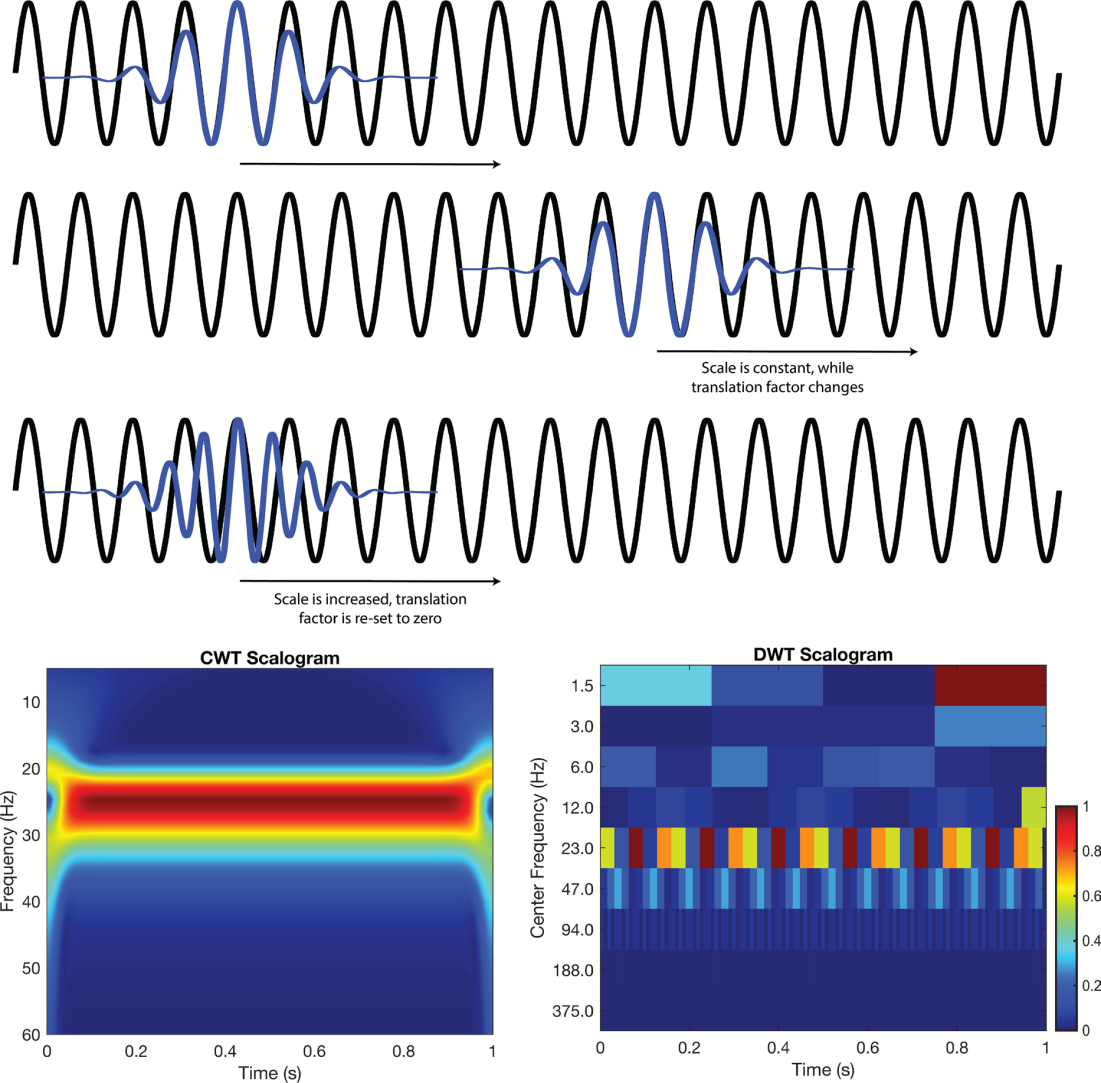
A 25 Hz sinusoid (black) is analyzed using Morlet daughter wavelets (blue). In the first two rows the daughter wavelet’s scale is fixed, giving a frequency of 25 Hz; as the daughter wavelet translates across the signal it remains perfectly in‐phase, yielding high correlations and thus energy responses. In the third row, the daughter wavelet’s scale is dilated (scale ↑) shifting its frequency to 27 Hz, resulting in the visible mismatch and thus lower correlation/energy response. The CWT scalogram (bottom left) therefore shows a bright, continuous red band at 25 Hz with a lower energy orange band just below at 27 Hz. Similarly, the DWT scalogram (bottom right) captures the same general pattern, albeit with discrete time–frequency blocks

Conceptually, the CWT is the convolution of a mathematical function, termed the mother wavelet, and the signal of interest. As shown in Fig. 1, the mother wavelet is shifted in time and scaled in frequency (e.g., compressed or dilated), which are often referred to as daughter wavelets. For example, the top two rows of Fig. 1 illustrate the daughter wavelet shifted in time (at time 0 and time 1). Once the daughter wavelet has completed sequential shifting and convolutions across the signal, it is scaled to new frequency (thus, new daughter wavelet), and beginning at time 0 as demonstrated in the third row. This process is iteratively repeated to assess how similar the daughter wavelets of different time and dilation are to all portions of the signal. The result is a plot of energy in the signal at a given time–frequency, as shown in the bottom row of Fig. 1 (left), and is typically referred to as a scalogram.

## An effective balance of efficiency and detail: discrete wavelet transform

Although the CWT can effectively describe the ERG, this method is computationally inefficient, as it performs redundant scales and shifts. In contrast, the DWT operates on discrete scales and dyadically down samples (powers of two), balancing efficiency while capturing essential signal features. Thus, the DWT evaluates only discrete frequencies and times.

The DWT follows a hierarchical process, where the signal is first passed through a high pass filter, and low pass filter. The outputs of these filters are then dyadically down sampled. This ultimately yields two bands of frequency content, with the high pass filter retaining an upper band called detail coefficients, and the low pass filter retaining a lower band called approximation coefficients. The approximation coefficients again go through this process, filtering through a high pass filter and low pass filter and are then down sampled. This iterative process of filtering and down sampling continues until down sampling can no longer take place, or a predetermined level is achieved. An example of DWT decomposition can be found in Fig. 1 (bottom right). The plot is similar to the CWT (bottom left), but the energy is plotted in discrete frequency-time bins.

Not only does the DWT offer computational efficiency thanks to its hierarchical decomposition, it is a reasonable compromise of all time, frequency, and time–frequency methods. This is because as each level yields a distinct set of approximation and detail coefficients, one can easily threshold coefficients from specific levels. Specifically, this can be applied to lower levels where there is unwanted noise (high frequency detail coefficients), or correct baseline drift (low frequency approximation coefficients). This multifaceted approach enables maximal physiological signal preservation while also easing the process of artifact removal. Additionally, interpreting the DWT numerical results are less subjective and straight forward compared to the CWT scalogram, as each energy index or coefficient relates to a specific time–frequency band.

This discrete, block-like representation often makes it easier to compare quantitative values across multiple subjects or conditions, facilitating robust statistical analysis and clear-cut clinical or research interpretations.

## Considerations and applications

For both the CWT and DWT, there are two important considerations: 1) wavelet analysis is affected by reduced reliability at the beginning and end of the signal. The wavelet cannot fully overlap the beginning and end of the signal, resulting in a region where energy estimates may be less accurate. Often, the signal is “padded” to minimize artifacts in the CWT that arise at the beginning and end of the signal. This may not be necessary for recordings that contain extended pre- or post-stimulus segments in the recording (e.g., baseline pre-stimulus recording). 2) The choice of mother wavelet is dependent on a variety of factors for both methods. In the DWT: Filter length influences temporal and spectral resolution, with shorter lengths yielding higher temporal and lower spectral resolution, and vice versa for longer length filters. Higher number of vanishing moments are better suited for removing low-order polynomial trends and capturing abrupt changes yet result in longer filter lengths. Orthogonal wavelets (Haar, Daubechies-dbN, Symlets-symN, Coiflets-coifN) yield non-redundant energy-preserving coefficients, and are thus well suited for feature extraction, while biorthogonal wavelets maintain linear-phase symmetry. Though the latter prevent phase distortion, the transform is no longer energy-orthonormal. Short, fairly symmetric orthogonal wavelets like sym2 balance these factors well for ERG analysis [12]. Like filter length modulation in the DWT, the CWT time-bandwidth product (i.e., the number of oscillatory cycles contained in the wavelet) can be adapted to improve either frequency or temporal resolution, at the cost of the other. For both DWT and CWT, selecting a mother wavelet whose form mirrors the signal’s own morphology enables the transform to better capture the underlying physiological energy patterns with maximal fidelity. Previous studies have utilized the Mexican Hat wavelet for its close match to typical ERG waveform profiles, whereas Morlet wavelets are often used to isolate high-frequency OPs in pathological ERGs [13].

**Fig. 2:**
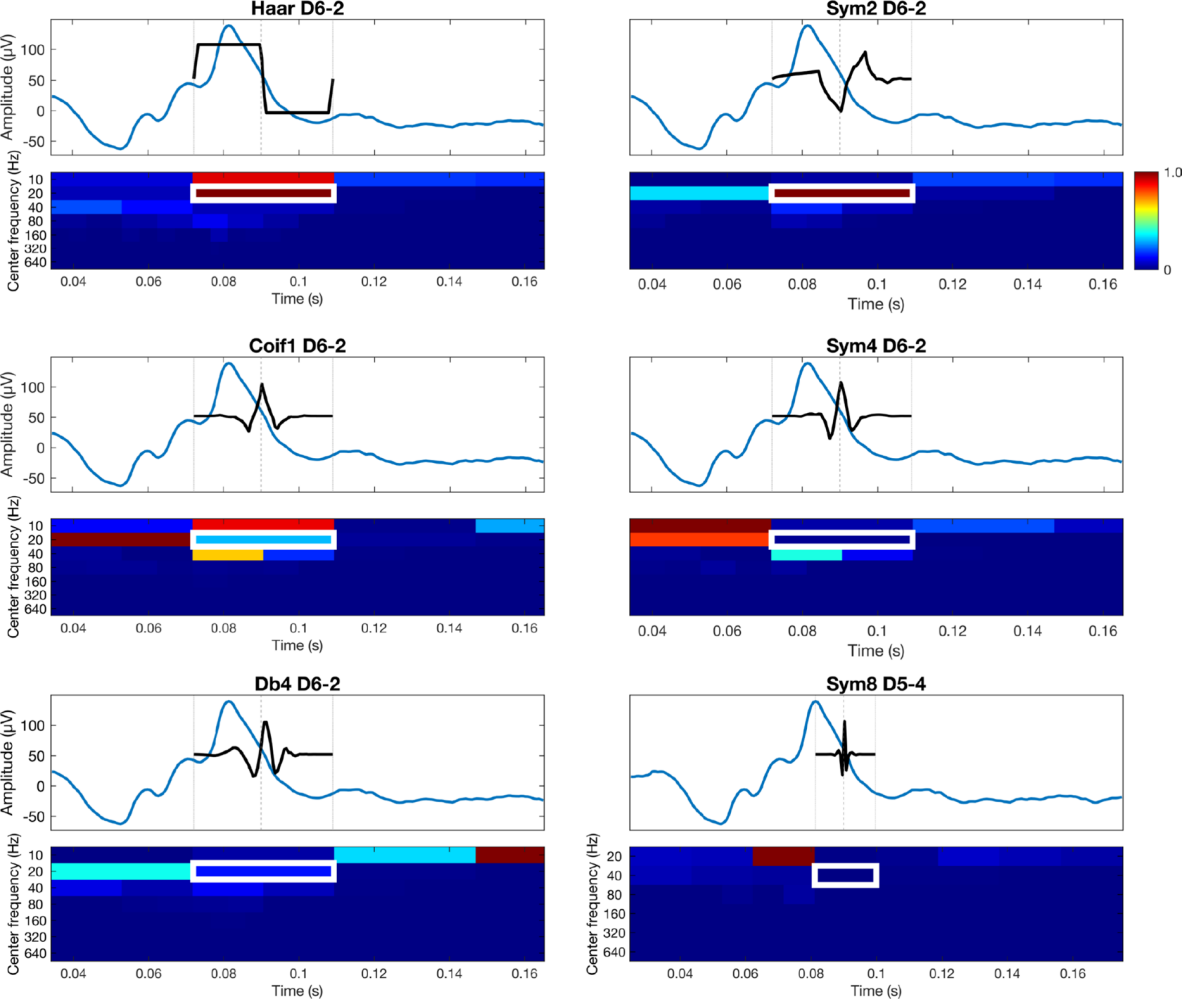
Six wavelet families are shown including Haar D6-2 (Decomposition level, D6—Index, 2), sym2 D6-2, coif1 D6-2, sym4 D6-2, db4 D6-2 and sym8 D5-4. For each panel, the top trace represents the time-domain waveform with the black curve denoting the scaled and shifted mother wavelet corresponding to the highlighted index; the bottom plot displays the DWT scalogram labeled by center frequency (Hz). Each signal window was trimmed and shifted so that the selected index was centered at 0.09 s (white rectangle), ensuring that all wavelets analyzed the same portion of the b-wave downslope. Energies were unit-normalized (0–1). Haar and sym2 most closely resembled the local waveform transition, producing stronger and more focal energy localization, whereas coif1, db4, sym4 and sym8 yielded broader or weaker distributions

We demonstrate the influence mother wavelet selection can have on the resultant WT in Fig. 2, using six families: haar, coif1, db4, sym2, sym4 and sym8, with the latter three representing members of the same wavelet family with progressively longer filter lengths. Each signals window was trimmed and shifted so that the white outlined energy index in each scalogram was centered at 0.09 s, ensuring all wavelets analyzed the same portion of the photopic b-wave. Despite subtle differences in morphology across families, the haar, and sym2 wavelets were best suited for capturing the localized energy of the ascending b-wave, as their shapes most closely resemble the transition of this region. The haar wavelet is entirely orthogonal and has shortest possible basis, making it highly time-localized and computationally efficient. Here the sym2 wavelet is effectively a mirror-image of the signal’s deflection; however, because wavelet energies are computed from the squared magnitude of the convolution coefficients, this inversion yields large energy localization (in contrast to large negative convolution coefficients). Mother wavelets that differ substantially in structure from this region, like that of coif1, db4, sym4 and sym8, yielded the expected lower energy indices.

In the previously mentioned work by Gauvin et al. forming the 40b:20b metric, this measure yielded values of 1.05 ± 0.06 (Normal), 2.01 ± 0.30 (complete congenital stationary night blindness, CSNB), and 0.43 ± 0.06 (congenital post-receptoral pathway anomaly/incomplete CSNB) [7, 12]. This ratio is especially useful when ON–OFF protocols are not available and can be used in parallel to establish concordance. As molecular diagnoses continue to diversify, objective pathway-resolved metrics may provide a useful bridge between genotype and function. This includes verifying whether a variants expression is consistent with inner-retinal vs photoreceptor involvement. Notably, DWT-derived OFF metrics may better capture the activity of OFF-bipolar cells than the i-wave [14]. Other work has shared cohort differences in patients with diabetic retinopathy [15], retinitis pigmentosa [16], glaucoma [17], and macular dystrophy [18]. However, validation with external datasets for each is needed before further integration can truly be considered.

Both CWT and DWT lend well toward clinical decision systems and machine learning pipelines while offering different advantages. For example, the CWT produces time–frequency images (scalogram heatmaps) that can be used as input for deep learning (DL), and is especially well suited for smaller datasets where image-based architectures can learn spatial energy patterns. Kulyabin et al. utilized multiple CWT scalograms with different mother wavelets in a visual transformer network for the task of classifying healthy vs unhealthy recordings [19]. Alternatively, the individual time–frequency indices provided by DWT can be easily utilized as interpretable features for statistical or traditional machine-learning classification when larger sample sizes are available. Constable et al. applied this approach to classify neurodevelopmental cohorts (ASD, ADHD, controls) using DWT-derived energy bands, integrating wavelet features into ensemble learning models producing balanced accuracies as high as 87% [20].

## Case study: applying fourier and wavelet methods to healthy ERGs

In the following section, we demonstrate the implementation and utility of WT methods in assessing full-field ERG (ffERG) recordings. Code and instructions to perform this same CWT and DWT analysis are available in the Code Availability section. Analysis was performed on a right-eye example recording from a healthy 32-year-old male.

Recordings were from the patient’s right eye using a DTL electrode. All signals were passed through a 0.3–300 Hz Butterworth band pass filter. Flash responses were sampled at 2,000 Hz. These signals are available in the Data Availability section. Rather than plotting the power spectrum, the amplitude (square root of the power) of each waveform was obtained (second column). All signals were detrended using the MATLAB detrend function, to remove DC offset prior to analysis.

**Fig. 3:**
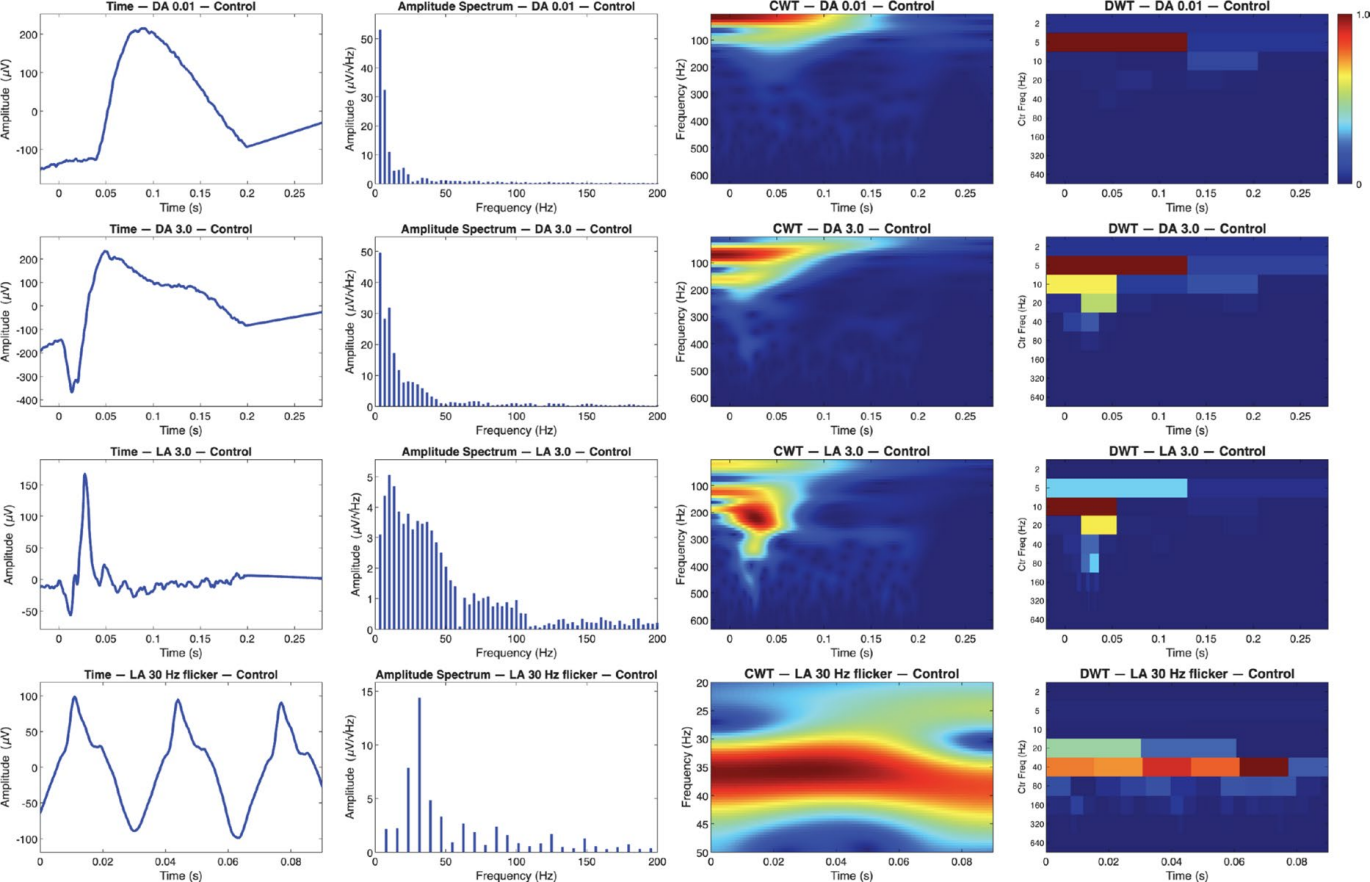
Full‐field ERG recordings from a healthy subject across four ISCEV standard recordings: DA 0.01 cd s/m^2^ (rows 1), DA 3.0 cd s/m^2^ (rows 2), LA 3.0 cd s/m^2^ (rows 3) and LA 30 Hz flicker (rows 4). Each row presents the time‐domain waveform, corresponding amplitude spectrum, and both continuous wavelet transform (CWT) and discrete wavelet transform (DWT) scalograms

Looking at the DA 0.01 cd s/m^2^ flash in Fig. 3 (row 1), the signal reaches a large, smooth b-wave peak at 0.08 s, consistent with a healthy rod-mediated response. The amplitude spectrum shows most of the energy is concentrated blow 50 Hz, with gradual tapering toward higher frequencies. The CWT scalogram mirrors this pattern, displaying high time–frequency energy localized early in the trace that coincides with the b-wave peak. Similarly, DWT has low-frequency activity near 5 Hz, aligning with slow temporal dynamics of the scotopic response.

In the DA 3.0 cd s/m^2^ condition (Fig. 3, row 2), the recording shows a defined a wave at 0.01 s, followed by a b-wace peaking near 0.05 s. The amplitude spectrum again demonstrates strong low-frequency components within 5–15 Hz, corresponding to the a–b range. The CWT demonstrates distinct regions of high energy aligning temporally with both wave deflections, while DWT shows distributed energy across 5, 10, 20 Hz center frequency bands.

Under the LA 3.0 cd s/m^2^ flash (Fig. 3, row 3), the waveform displays an a-wave trough followed by a b-wave, characteristic of a strong cone-mediated response. The amplitude spectrum is broader than both of the previous scotopic conditions with energy extending beyond 100 Hz. In the CWT, two distinct peaks of localized energy appear around 100 Hz and 200 Hz. Conversely, the DWT reflects the amplitude spectrum with strong activity in the range of 10, 20 and 40 Hz indices, reflecting faster temporal structure of cone system responses.

Lastly the LA 30 Hz flicker cd s/m^2^ (Fig. 3, row 4) exhibits evenly spaced oscillations near 100 µV, representing an established and periodic cone-driven response. The amplitude spectrum shows a dominant 30 Hz fundamental with clearly defined harmonics. In the CWT this steady high-energy band is constant across at ~ 30 Hz, while the DWT demonstrates prominent activity in this same region at the 20 Hz and 40 Hz center-frequency bins.

## Conclusion

As demonstrated, advanced signal processing techniques like the CWT and DWT provide qualitative and quantitative insights for ERG analysis. While time-domain features offer some pathological insight, a complete picture can be obtained with these additional time–frequency based measures. However, no single method is superior to another, and the analytical choice of approach is dependent on a variety of factors including stimulus, pathology, or feature of interest. Ultimately, integrating wavelet-based techniques into routine ERG analysis may significantly enhance diagnostic accuracy and provide deeper insight into retinal function and pathology.

## Data Availability

The data utilized in this study is made available in the following GitHub repository.

## Appendix

### Continuous wavelet transform

The continuous wavelet transform (CWT) is defined by:

1 $$CWT_{j, k} = \mathop \sum \limits_{n = 0}^{N - 1} x_{n} \cdot \psi_{j, k}^{*} \left( n \right) = \mathop \sum \limits_{n = 0}^{N - 1} x_{n} \cdot \psi_{j, k}^{*} \left( {\frac{n - k}{{2^{j} }}} \right)$$

where

- ${x}_{n}$ is the original signal,
- $\psi (n)$ is the mother wavelet,
- $j$ and $k$ control scaling (frequency) and shifting (time), respectively
- $*$ denotes complex conjugation.

Each mother wavelet $\psi$ has a central frequency ${F}_{c}$. Scaling by a factor $a$ yields a daughter wavelet with a pseudo-frequency ${F}_{a}$:

2 $${F}_{a}=\frac{{F}_{c}}{a}$$

In practice, $a= {2}^{j}$. The local wavelet energy is:

3 $${E}_{j, k} = {\left|{CWT}_{j, k}\right|}^{2}$$

The mother wavelet’s central frequency ${F}_{c}$ defines its fundamental sinusoidal structure, so when you scale and slide ($j$ and $k$, respectively) $\psi (n)$ across the signal ${x}_{n}$, those segments of ${x}_{n}$ that most closely match $\psi \left(n\right)$ at that pseudo frequency exhibit high correlation (and hence high energy, ${E}_{j, k}$), whereas poorly matched regions produce near‐zero coefficients.

### Discrete wavelet transform

The DWT is defined as follows:

4 $$DWT_{j, k} = \mathop \sum \limits_{n = 0}^{N - 1} x_{n} \cdot \psi_{j, k}^{*} \left( n \right) = \mathop \sum \limits_{n = 0}^{N - 1} x_{n} \cdot \psi_{j, k}^{*} \left( {\frac{{n - k \cdot 2^{j} }}{{2^{j} }}} \right)$$

In practice, the DWT is implemented by passing the original signal through the following steps:

1. A high-pass filter and low-pass filter yielding detail and approximation coefficients.
2. Both sets of coefficients are then down sampled by a factor of two.
3. Approximation coefficients are selected and iteratively processed through steps 1–3 until decomposition can no longer takes place.

Mathematically, each decomposition level is defined as $j$:

5 $$A_{{j_{n} }} = \mathop \sum \limits_{k = 0}^{N - 1} A_{j - 1, k} L_{{n - 2^{j} k}}$$

6 $$D_{{j_{n} }} = \mathop \sum \limits_{k = 0}^{N - 1} A_{j - 1, k} H_{{n - 2^{j} k}}$$

Energy of these approximation and detail coefficients are calculated as:

7 $$E_{{A_{j} }} = \mathop \sum \limits_{k = 0}^{N - 1} \left| {A_{j, n} } \right|^{2}$$

8 $$E_{{D_{j} }} = \mathop \sum \limits_{k = 0}^{N - 1} \left| {D_{j, n} } \right|^{2}$$

## Code availability

Code to execute discussed analysis is available in the following GitHub repository.
